# Deep Learning Enabled Ultrafast Optical Spectral Phase Retrieval via Unbalanced‐Intensity Autocorrelation

**DOI:** 10.1002/nap2.70144

**Published:** 2026-05-21

**Authors:** Tae‐In Jeong, Eunji Choi, Mengkun Liu, Seungchul Kim, Alexander Gliserin

**Affiliations:** ^1^ Department of Opto‐Mechatronics Engineering Pusan National University Busan South Korea; ^2^ Department of Cogno‐Mechatronics Engineering Pusan National University Busan South Korea; ^3^ Department of Physics and Astronomy Stony Brook University Stony Brook New York USA; ^4^ National Synchrotron Light Source II, Brookhaven National Laboratory Upton New York USA

## Abstract

Complete characterization of ultrafast optical fields is essential for deploying femtosecond pulse sources in integrated photonic systems, yet conventional techniques rely on spectrally resolved detection and iterative phase retrieval. Recently, phase‐enabled nonlinear gating with unbalanced intensity (PENGUIN) showed that spectral phase information is preserved in an unbalanced nonlinear interferometric autocorrelation (IAC) enabling field retrieval without spectrally resolved detection. However, the original iterative reconstruction algorithm is only reliable for pulses near the Fourier‐transform limit. To overcome this dispersion limitation, we introduce DeepPENGUIN, a deep‐learning framework for direct spectral phase inference from unbalanced‐intensity IAC signals in the frequency domain without iterative retrieval. The model is trained on large‐scale synthetic data through physics‐informed supervised learning with spectral and mathematical constraints, providing a robust solution for the one‐dimensional phase retrieval problem for which no general inversion framework has been established. Validation using real‐world few‐cycle (8 fs) pulses demonstrates reliable phase retrieval over a large dispersion range from 0 to 200 fs^2^. A quantitative noise robustness analysis shows performance comparable to ptychographic frequency‐resolved optical gating across a broad range of noise levels. The feedforward inference architecture enables real‐time pulse reconstruction with millisecond‐level latency, providing a scalable route toward integrated ultrafast pulse diagnostics and feedback control.

## Graphical abstract

Phase‐enabled nonlinear gating with unbalanced intensity (PENGUIN) enables reconstruction of the complete ultrafast optical field from one‐dimensional nonlinear interferometric autocorrelation traces. Combined with deep learning, the DeepPENGUIN framework enables real‐time spectral phase retrieval without spectrally resolved detection or iterative algorithms while remaining robust under strong dispersion, highlighting its potential for compact and integrated ultrafast photonic systems.

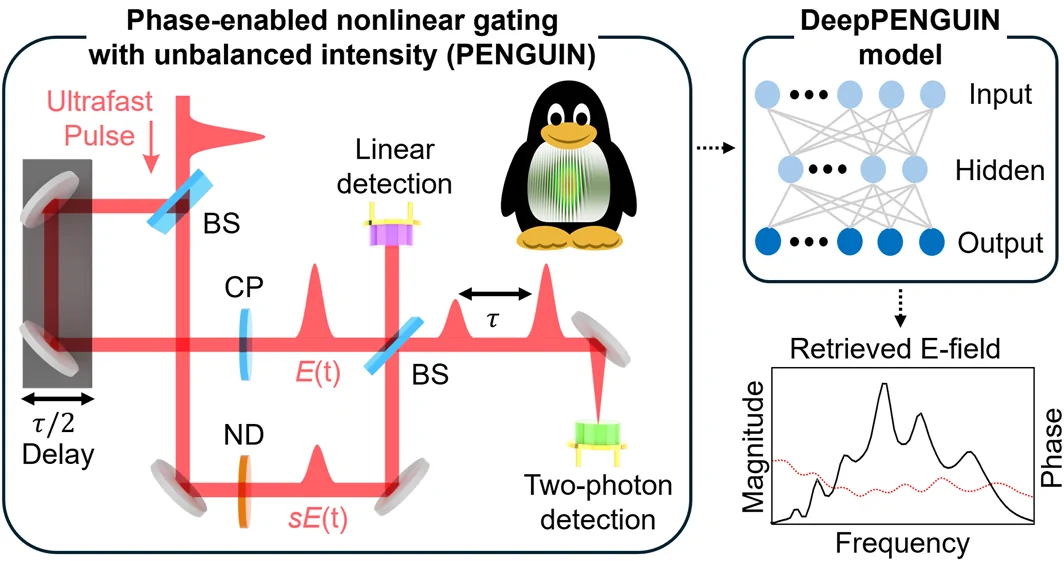

## 1 Introduction

Silicon photonic integration is advancing rapidly as a platform for high‐bandwidth communication, large‐scale optical information processing, and emerging quantum technologies [1, 2, 3, 4, 5]. Although continuous‐wave lasers have been used as the primary on‐chip optical sources, recent demonstrations of femtosecond pulse generation using thin‐film lithium niobate and soliton formation in micro‐ring resonators have expanded the available operating regimes [6, 7, 8]. These on‐chip ultrafast pulses enable parallel optical communication and advances in nonlinear photonic computing and quantum photonics by leveraging the broadband frequency content and high peak intensity inherent to ultrafast optical pulses [9, 10, 11].

To fully exploit these ultrafast sources in on‐chip systems, the optical field must be directly characterized within the photonic circuit [12, 13, 14]. Dispersion and nonlinear effects in the material modify the spectral phase and thus the temporal profile of ultrafast pulses. Accurate knowledge of these field properties is therefore essential for monitoring and controlling the stability and performance of integrated communication and quantum photonic systems [15, 16, 17, 18]. In photonic computing in particular, pulse shaping is used to implement computational primitives, and the resulting changes in temporal structure and phase must be tracked, since these distortions directly govern computation accuracy [19, 20, 21, 22, 23]. Access to the full electric field is therefore essential for enabling performant, reliable and controllable ultrafast silicon photonic platforms.

A variety of techniques have been developed for complete characterization of ultrashort optical pulses. Frequency‐resolved optical gating (FROG) reconstructs the pulse field from spectrally resolved nonlinear autocorrelation measurements (spectrograms) through an iterative solution of the two‐dimensional phase retrieval problem, whereas spectral phase interferometry for direct electric‐field reconstruction (SPIDER) enables direct spectral phase extraction from spectrally sheared interferograms without delay scanning [24]. The modified interferometric field autocorrelation (MIFA) method demonstrated non‐iterative spectral phase recovery from two nonlinear autocorrelation traces acquired without the need for spectrally resolved detection using a specially engineered nonlinear waveguide with narrowband phase matching [25]. More recently, optimization‐assisted methods based on programmable pulse shaping and optical chirplet‐domain reconstruction have been introduced for ultrafast field recovery by controlling the spectral phase to yield a Fourier‐transform‐limited pulse [26]. These methods have significantly advanced ultrafast optical metrology, but practical trade‐offs remain regarding implementation complexity, measurement flexibility, and computational cost.

Data‐driven approaches for ultrashort pulse characterization have recently emerged as a promising alternative to conventional iterative phase retrieval techniques [27, 28]. In particular, Zahavy et al. demonstrated, using FROG measurements, that deep neural networks can successfully learn the mapping between spectral‐domain representations and the corresponding ultrashort pulse phase, establishing an important foundation for machine‐learning assisted ultrafast metrology [29]. This work highlighted the potential of neural networks to infer phase‐sensitive information directly from a measured optical signal without relying on iterative solvers.

Several approaches have been explored for on‐chip characterization of ultrashort pulses [12]. One approach is to generate harmonics via a nonlinear medium inside the photonic circuit and measure the resulting signal externally [14, 30]. More recently, this concept has been extended by applying FROG to the harmonic signal using an integrated nonlinear element together with an off‐chip spectrometer, which enabled complete pulse characterization [13]. These schemes, however, still rely on spectrally resolved detection, which increases fabrication complexity, limits low‐power signal sensitivity, and restricts real‐time operation [31, 32]. An alternative approach is to perform a nonlinear autocorrelation measurement directly on a chip utilizing two‐photon absorption (TPA) in semiconductor photodiodes, which does not require spectral resolution [33, 34]. Although this method enables fully integrated diagnostics, it does not provide spectral phase information and therefore cannot reconstruct the full electric field [35, 36].

Recently, it has been demonstrated that an unbalanced‐intensity nonlinear interferometric autocorrelation (IAC) breaks the time‐reversal symmetry of a conventional autocorrelation and allows the IAC signal to retain spectral phase information, which is otherwise lost in a conventional balanced autocorrelation [37]. This method, termed phase‐enabled nonlinear gating with unbalanced intensity (PENGUIN), enables the full reconstruction of the ultrafast optical field from the IAC measurement alone due to the phase information preserved within the signal, and requires no spectrally resolved measurements. However, the PENGUIN approach previously relied on an iterative field retrieval algorithm which performs best for pulses near the Fourier‐transform limit but becomes unstable in the high‐dispersion regime. Extending the practical applicability of this technique to strongly dispersed pulses, particularly those with large group delay dispersion (GDD), is essential for its scalability to integrated photonic circuits, where material dispersion plays a crucial role, as well as for applications that intentionally employ dispersed pulses, such as optical parametric chirped pulse amplification.

Here, to overcome the instability of the original PENGUIN algorithm in the high‐dispersion regime, we introduce a new analysis framework, DeepPENGUIN, a data‐driven deep learning model that infers the physical mapping through which the unbalanced‐intensity IAC signal encodes the spectral phase of the optical field. Unlike previous machine‐learning studies in ultrafast optical metrology that primarily accelerate or approximate already established retrieval frameworks based on two‐dimensional (spectrogram type) measurements, the present work addresses a fundamentally unresolved inverse problem of reconstructing the field from a one‐dimensional phase‐preserving IAC measurement. In a balanced autocorrelation, the loss of spectral phase information prohibits a unique inversion except for special cases, whereas the phase‐preserving unbalanced‐intensity IAC substantially constrains the solution space where a unique numerical solution (except for trivial ambiguities such as linear spectral phase) has been empirically shown for a wide range of practical cases [37] despite the lack of a mathematical proof. Within this premise, the DeepPENGUIN framework aims to provide accurate spectral phase inference by exploiting the constraints of the phase information contained within the unbalanced‐intensity IAC signal in addition to mathematical and spectral constraints. Due to the empirical nature of this approach, the results shown in this work do not necessarily imply universality, but rather a reliability of the model within the demonstrated large parameter space of practical pulses relevant to ultrafast optics experiments.

The model uses the unbalanced‐intensity IAC signal and the power spectrum of the pulse as its input and reconstructs the original electric field in the frequency domain without iterative solving. Large‐scale simulations spanning diverse amplitude and phase conditions show accurate field retrieval across a wide range of dispersion. To evaluate the performance of our deep learning model under experimentally realistic conditions, we used an 8 fs few‐cycle ultrafast optical pulse measured by second‐harmonic‐generation (SHG)‐FROG with a well‐established ptychographic retrieval algorithm as the reference electric field for the DeepPENGUIN framework. The results show that DeepPENGUIN overcomes the GDD‐related limitations of the original PENGUIN algorithm, enabling reliable reconstruction of pulses in the high‐dispersion regime far from the Fourier‐transform limit, demonstrated with a GDD of up to 200 fs^2^ in this work. Furthermore, a quantitative investigation of the noise robustness shows that its performance is comparable to that of ptychographic FROG. This demonstrates that complete ultrafast field characterization does not fundamentally require spectrally resolved detection, even in a high‐dispersion regime and realistic noise environment. Integrating DeepPENGUIN with an on‐chip nonlinear gating element, such as a TPA‐based photodiode array or a nonlinear waveguide, enables monolithic and real‐time electric‐field monitoring for ultrafast sources embedded within photonic circuits. This capability supports feedback control, adaptive operation, and coherent processing in integrated ultrafast photonic systems.

## 2 Methods

### 2.1 Principle of Phase‐Preserving Unbalanced‐Intensity IAC

The PENGUIN technique, as depicted in Figure 1, is described in detail elsewhere [37]. In short, it is based on a standard interferometric autocorrelator, shown here in a Mach‐Zehnder configuration (Figure 1a), which is modified by introducing an attenuation in one of the arms in order to reduce the amplitude of an ultrafast (complex‐valued) electric field, E(t), in that arm by a balance factor 0 < *s ≤* 1. This can be achieved either via a discrete attenuator such as a neutral‐density (ND) filter, by using beam splitters with different ratios, or by waveguides with different loss in a photonic circuit. With this modification, a nonlinear photodetector, such as a suitable PD to produce TPA photocurrent, then provides the unbalanced‐intensity IAC as a function of the autocorrelation delay *τ*. For a second‐order nonlinearity, such as TPA, the unbalanced‐intensity IAC signal is given by [37]:

(1) $$\begin{array}{c} I_{\text{IAC}}^{\left(2\right)}\left(\tau \right)=\int_{-\infty }^{+\infty }{\left|{\left(sE\left(t\right)+E\left(t-\tau \right)\right)}^{2}\right|}^{2}dt \end{array}.$$

**FIGURE 1:**
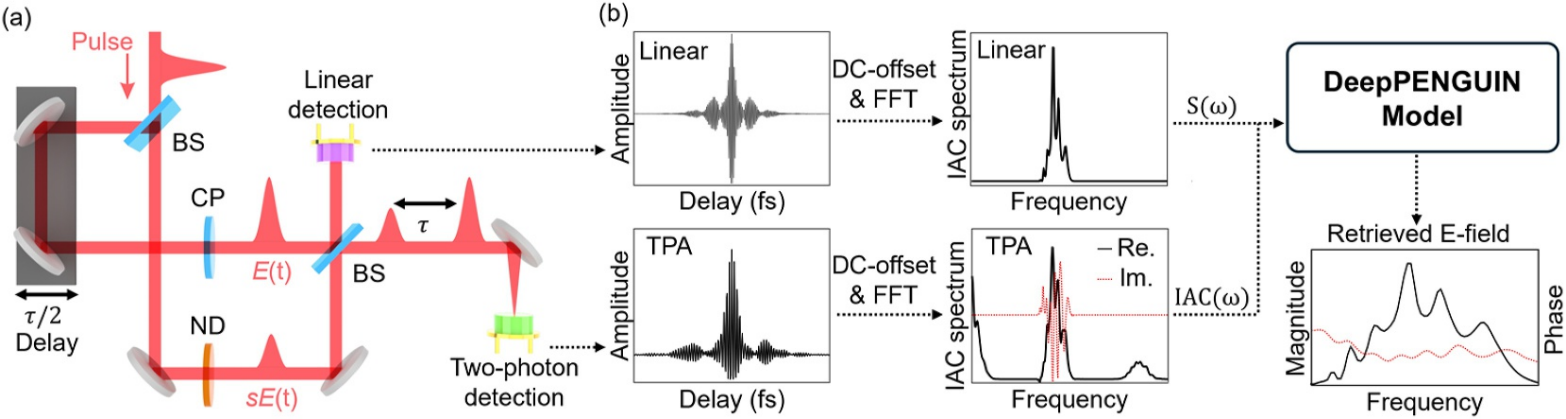
Schematic of the phase‐preserving unbalanced‐intensity nonlinear IAC for PENGUIN. (a) Unbalanced‐intensity interferometric autocorrelator using an attenuator (ND filter) in one of the arms of a Mach‐Zehnder interferometer to reduce the amplitude of an ultrafast electric field E(t) by a balance factor of *s* < 1. The TPA photocurrent from a nonlinear photodetector (green) records the second‐order unbalanced‐intensity IAC as a function of the delay *τ*. The optical power spectrum can be simultaneously measured via linear IAC (purple) at the second output of the interferometer. BS: beam splitter; CP: compensating plate for dispersion balancing. (b) Prediction flow of the DeepPENGUIN model. The measured time‐domain IAC signal is Fourier transformed into the frequency domain, and the real and imaginary parts of the complex signal, together with the measured power spectrum, S(ω), are provided as inputs to the DeepPENGUIN model. The network outputs the predicted spectral phase, which is combined with the measured power spectrum to reconstruct the complex electric field.

A balance factor of *s <* 1 breaks the time‐reversal symmetry of the IAC signal in Equation (1) and therefore preserves spectral phase information, which is otherwise lost in a balanced IAC. The phase‐preserving fundamental‐frequency component of the IAC signal can be extracted by spectral filtering and solved for the fundamental field, E(t), with a simple numerical algorithm [37]. This field retrieval approach is presented in detail in Supporting Information S1: Section 1 and Figure S1. In addition to nonlinear detection, the interferometric autocorrelator setup shown in Figure 1a also allows simultaneous linear detection of the interference signal, for example via a linear PD, which facilitates time‐domain spectroscopy of the pulse. The measured linear IAC signal is given by:

(2) $$\begin{array}{c} I_{\text{IAC}}^{\left(1\right)}\left(\tau \right)=\int_{-\infty }^{+\infty }{\left|sE\left(t\right)+E\left(t-\tau \right)\right|}^{2}dt \end{array}.$$

After removing the delay‐independent DC background contribution, the frequency‐domain representation of the mixed term in Equation (2) yields $2s{\left|E\left(\omega \right)\right|}^{2}$ via the convolution theorem, where E(ω)=F{E(t)} denotes the Fourier transformation of the electric field from the time domain into the frequency domain. Thus, the Fourier transform of the background‐free linear IAC signal is directly proportional to the optical power spectrum, $S\left(\omega \right)={\left|E\left(\omega \right)\right|}^{2}$. Furthermore, in a linear IAC, a balance factor of *s <* 1 only reduces the contrast between the time‐independent background and the interferometric signal but does not break the time‐reversal symmetry of the linear IAC, which enables simultaneous measurements of the optical power spectrum and the phase‐preserving nonlinear IAC. Note that in this paper, apart from Figure 1, the term IAC implicitly refers to the phase‐preserving unbalanced‐intensity nonlinear IAC as introduced in Equation (1), and the optical power spectrum obtained from the linear IAC in Equation (2) (or from an external spectroscopic measurement) is referred to as S(ω). The DeepPENGUIN framework uses both the unbalanced‐intensity nonlinear IAC signal and the power spectrum for spectral phase inference in the frequency domain, as illustrated in Figure 1b. The experimentally recorded IAC signal in the time domain is first Fourier transformed to obtain the complex IAC spectrum, and its real and imaginary parts, together with the power spectrum, S(ω), are provided as inputs to the DeepPENGUIN model. The network then outputs the predicted spectral phase, which is combined with the power spectrum to obtain the complex electric field.

### 2.2 Principle of the DeepPENGUIN Framework

The computational flow and internal mechanism of the DeepPENGUIN framework are illustrated in Figure 2. The input to the network consists of three experimentally accessible quantities: the real and imaginary parts of the complex IAC spectrum, and the optical power spectrum, $S\left(\omega \right)={\left|E\left(\omega \right)\right|}^{2}$ (Figure 2a). Since the IAC signal, $I_{\text{IAC}}^{\left(2\right)}\left(\tau \right)$, is measured in the time domain (Equation (1)), it is Fourier transformed into the frequency domain, which yields its complex spectrum whose real and imaginary parts are supplied to the network. The Cartesian representation of the IAC spectrum with real and imaginary parts is the more practical choice for training the model compared to the polar representation (Euler form) with magnitude and phase. Since the phase suffers from 2π discontinuities, it requires unwrapping in addition to appropriate scaling and weighting by the spectral magnitude to be used by the deep‐learning model, whereas the real and imaginary parts are dimensionally equivalent and do not require any relative scaling.

**FIGURE 2:**
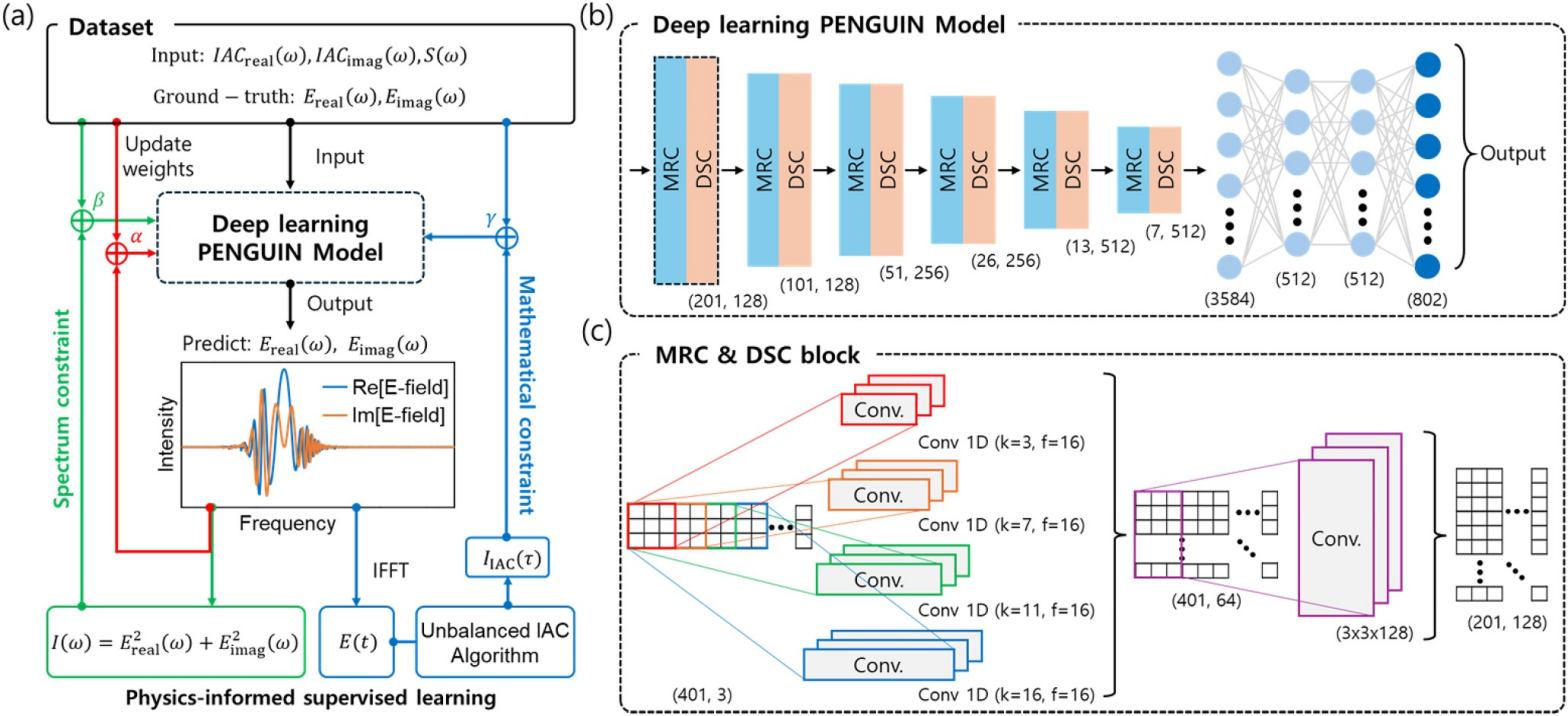
Principle and architecture of the DeepPENGUIN model. (a) Schematic of the model training: The time‐domain IAC signal is Fourier transformed and the real and imaginary parts of the resulting complex IAC spectrum, together with the power spectrum, S(ω), are provided as inputs to the DeepPENGUIN model, which is trained through physics‐informed supervised learning. (b) The DeepPENGUIN network consists of six sequential MRC‐DSC modules, where MRC extracts spectral features across different frequency scales and the DSC compresses them into compact spectral representations. The subsequent fully connected layers integrate long‐range spectral correlations and generate the reconstructed spectral phase outputs as cosϕ(ω) and sinϕ(ω). (c) Internal configuration of the first MRC‐DSC block, where parallel 1D convolutional filters with different kernel sizes capture multiscale spectral features, and a down‐sampling convolution reorganizes them into a more compact representation.

The DeepPENGUIN model is designed to predict the spectral phase of the optical field at each frequency bin. The complex electric field can be expressed in the frequency domain as $E\left(\omega \right)=\sqrt{S\left(\omega \right)}e^{i\phi \left(\omega \right)}$, where $S\left(\omega \right)={\left|E\left(\omega \right)\right|}^{2}$ is the measured power spectrum and ϕ(ω) is the unknown spectral phase. Since the spectral magnitude $\sqrt{S\left(\omega \right)}$ is provided from a separate measurement (see Figure 1b) as an external constraint, the task of DeepPENGUIN is reduced to predicting only the phase function. Direct regression of the phase angle is often unstable because the phase is periodic and contains 2π discontinuities. Instead, the model internally represents the frequency‐domain field in Cartesian form consistent with the form of the IAC spectrum used as an input. Thus, for each frequency bin, the network outputs two unconstrained real‐valued functions, a(ω) and b(ω), representing the real and imaginary components of the field, E(ω)=a(ω)+ib(ω). Since only the phase is returned by the model, which is the angle between a(ω) and ib(ω) in the complex plane, the magnitude of the complex‐plane vector spanned by these outputs is subsequently normalized to unity,

(3) $$\begin{array}{c} \left(\hat{a}\left(\omega \right),\hat{b}\left(\omega \right)\right)=\frac{\left(a\left(\omega \right),b\left(\omega \right)\right)}{\sqrt{a^{2}\left(\omega \right)+b^{2}\left(\omega \right)}}, \end{array}$$

resulting in ${\hat{a}}^{2}\left(\omega \right)+{\hat{b}}^{2}\left(\omega \right)=1$. This unit normalization decouples the phase information from the spectral magnitude, which is supplied as an external constraint, while maintaining a continuous Cartesian representation of the spectral phase, thus avoiding ambiguities associated with direct phase regression [38]. The normalized components $\hat{a}\left(\omega \right)$ and $\hat{b}\left(\omega \right)$ directly correspond to the cosine and sine of the optical field's spectral phase, that is, $\hat{a}\left(\omega \right)=\cos\;\phi \left(\omega \right)$ and $\hat{b}\left(\omega \right)=\sin\;\phi \left(\omega \right)$, respectively, according to the Euler identity, whereas its spectral magnitude is given by the measured power spectrum, S(ω):

(4) $$\begin{array}{c} \text{Re}\left[E\left(\omega \right)\right]=\sqrt{S\left(\omega \right)}\;\cos\;\phi \left(\omega \right),\text{Im}\left[E\left(\omega \right)\right]=\sqrt{S\left(\omega \right)}\;\sin\;\phi \left(\omega \right) \end{array}.$$

Although the model internally uses a Cartesian representation of all complex quantities (as real and imaginary parts), the reconstructed phase is easily obtained from the output functions $\hat{a}\left(\omega \right)$ and $\hat{b}\left(\omega \right)$ via $\phi \left(\omega \right)=\arctan\left(\hat{b}\left(\omega \right)/\hat{a}\left(\omega \right)\right)$. By using the power spectrum as a constraint, the spectral magnitude of the reconstruction is guaranteed to match the measured spectrum and the deep learning network is focused solely on spectral phase prediction. The training process is guided by three physics‐based loss functions *L*:

(5) $$\begin{array}{c} L_{\text{total}}=L_{\text{Field}}+L_{\text{IAC}}+L_{\text{Spec}}, \end{array}$$

where $L_{\text{Field}}$ penalizes the deviation between the predicted, $\hat{E}$, and ground‐truth electric fields, $E_{\text{true}}$:

(6) $$\begin{array}{c} L_{\text{Field}}={\left\|\hat{E}-E_{\text{true}}\right\|}^{2} \end{array},$$

$L_{\text{IAC}}$ is the error between the predicted and true IAC signals:

(7) $$\begin{array}{c} L_{\text{IAC}}={\left\|I_{\text{IAC}}\left(\hat{E},\hat{s}\right)-I_{\text{IAC},\text{true}}\right\|}^{2} \end{array},$$

where the predicted IAC is computed from the predicted electric field $\hat{E}$ and the given balance factor $\hat{s}$ according to Equation (1). The spectral power error, $L_{\text{Spec}}$, ensures consistency between the reconstructed and measured power spectrum, $S_{\text{true}}$:

(8) $$\begin{array}{c} L_{\text{Spec}}={\left\|{\left|\hat{E}\right|}^{2}-S_{\text{true}}\right\|}^{2} \end{array}.$$

Together, these three loss terms constrain the machine learning process from complementary perspectives. $L_{\text{Field}}$ directly supervises the predicted electric field (Equation (4)) using the ground‐truth label. Using the full field rather than just the spectral phase naturally introduces a weighting at each frequency bin (via the magnitude) and thus eliminates loss contribution from regions with low spectral magnitude where the phase is not well‐defined. $L_{\text{IAC}}$ enforces mathematical consistency by requiring that the IAC signal recalculated from the predicted electric field through Equation (1) agrees with the true IAC signal. $L_{\text{Spec}}$ constrains the spectral magnitude to remain consistent with the measured power spectrum, allowing the network to focus primarily on spectral phase prediction. Although the spectral consistency is definitionally satisfied by Equation (4), maintaining this loss term during early training improves convergence stability and suppresses outliers. In this framework, the proposed approach constitutes a physics‐informed supervised learning method, in which machine learning of the dependencies between the underlying spectral phase and the resulting unbalanced‐intensity nonlinear IAC signal is augmented by physically enforced constraints for measurement consistency. Accordingly, the inverse problem of phase retrieval is not solved over an unrestricted function space, but over a constrained manifold defined by measured observables and physically realizable pulses, as well as the pulse parameter space covered by the training data.

The sequence of the DeepPENGUIN model is presented in Figure 2b, which shows an encoder composed of six sequential multi‐resolution convolution (MRC) and down‐sampling convolution (DSC) modules [29, 39, 40]. Each MRC block applies several 1D convolutions with different kernel sizes in parallel to extract spectral features across multiple frequency scales. The subsequent DSC block reduces the spectral dimension through strided convolution while expanding the channel depth, allowing the network to form progressively more compact and expressive spectral representations as the layers deepen. After the MRC‐DSC module, the resulting feature map is flattened and processed by two fully connected layers, which integrate long‐range spectral dependencies and generate the outputs cosϕ(ω) and sinϕ(ω) of the reconstructed spectral phase. Additionally, the internal structure of an individual MRC‐DSC block is illustrated in Figure 2c, where the MRC paths and the subsequent DSC path operate together to transform the spectral representation. Further architectural details and the underlying design considerations of the DeepPENGUIN model, including activation function choices, multi‐resolution feature extraction strategy, and phase‐constrained output representation, are provided in Supporting Information S1: Section 2 [41, 42, 43]. This feedforward prediction process eliminates iterative phase retrieval and enables real‐time reconstruction using solely the measured IAC signal and the power spectrum. Unlike conventional iterative retrieval methods that repeatedly optimize the electric field for each individual measurement, DeepPENGUIN learns the inverse mapping between the spectral phase and the resulting IAC once during training and applies it directly to measurements of unknown pulses through a single feedforward pass. In practice, the inference latency of the DeepPENGUIN model is approximately 29 ms for a single IAC trace (median value over 300 runs), corresponding to an update rate of about 34 Hz. The 99th‐percentile latency remains below 36 ms, confirming stable real‐time operation. The benchmark was performed on a single GPU (Nvidia RTX 3060) with the model fully loaded into memory, excluding data loading and visualization overhead. This inference speed is substantially faster than conventional iterative phase retrieval algorithms for two‐dimensional spectrogram data (e.g., FROG) and is sufficient for online pulse shape monitoring and real‐time experimental feedback in ultrafast optical systems.

### 2.3 Preparation of Training Data

The training dataset for DeepPENGUIN was generated in the frequency domain by systematically varying both the spectral magnitude and phase of the complex electric field to cover a broad range of physically realistic ultrafast pulse conditions. The spectral magnitude was constructed with one to six Gaussian peaks, where the number of peaks, individual peak positions, full width at half maximum, and relative amplitudes were independently swept within predefined ranges. This procedure produced a diverse set of multi‐peak spectral profiles spanning narrowband to broadband conditions that are common for ultrafast pulses (see Supporting Information S1: Figure S2a).

The spectral phase was generated using a Fourier‐series based approach [29]. Random coefficients were assigned to discrete low‐frequency Fourier components under a Hermitian symmetry constraint, ensuring that the inverse Fourier‐transformed electric field remains real‐valued, as required by physical causality. For the training dataset used in this work, the maximum Fourier mode *K* was systematically swept over the values *K* = 300, 350, 400, 450, 500, and 550, allowing controlled variation of the correlation length and structural complexity of the generated phase function, with random coefficients [0…1] as relative weights of the modes. After inverse Fourier transformation, the resulting phase was normalized and scaled to a fixed maximum phase value, with the scaling factor swept in the range of 2, 3, 4 and 5 radians, thereby defining the absolute strength of the spectral phase modulation. This procedure ensures continuous and physically meaningful phase behavior while avoiding unphysical rapid phase oscillations. By sweeping different combinations of maximum Fourier mode *K* and scaling factors, the dataset encompasses a wide variety of phase profiles ranging from slowly varying to highly structured phase functions. To account for dispersion, additional quadratic spectral phase corresponding to GDD was applied directly in the frequency domain according to

(9) $$\begin{array}{c} \phi_{\text{GDD}}\left(\omega \right)=\frac{1}{2}\phi_{2}{\left(\omega -\omega_{0}\right)}^{2} \end{array},$$

where $\phi_{2}$ denotes the GDD value and $\omega_{0}$ is the central frequency. Individual DeepPENGUIN models were trained for discrete GDD values of 0, 10, 20, 30, 40, 50, 60, 70, 80, 100, 150, and 200 ${\text{fs}}^{2}$. For each GDD condition, one million synthetic pulse realizations were generated to ensure sufficient coverage of the magnitude‐phase parameter space. For all training data, the balance factor of the IAC was fixed at *s* = 0.5. The corresponding IAC signals were then computed from the synthesized electric fields and used as inputs for training DeepPENGUIN with the respective power spectra (see Supporting Information S1: Figure S2b,c). This data generation strategy ensures that both the magnitude and phase components of the training set remain physically realistic while providing sufficient diversity for robust data‐driven learning across a wide range of ultrafast pulse conditions.

## 3 Results

### 3.1 Validation With Simulation Data

The DeepPENGUIN model was validated on a set of synthetic electric fields that were not included during training to evaluate the performance with arbitrary pulse shapes. The test set consisted of pulses with multiple Gaussian spectral peaks combined with a random phase parametrized by a low‐order Fourier series, enabling controlled variation of both the magnitude structure and phase complexity. Representative examples are shown in Figure 3 for cases containing three and four spectral peaks. For each case, the synthesized complex electric field in the frequency domain was Fourier transformed into the time domain to compute the IAC signal according to Equation (1) with *s =* 0.5, which was then used as the model input along with the corresponding reference power spectrum, $S\left(\omega \right)={\left|E\left(\omega \right)\right|}^{2}$. The DeepPENGUIN model accurately predicted the spectral phase, from which the full complex electric field was reconstructed in the frequency domain by combining it with the reference power spectrum. The resulting field was subsequently used to calculate the corresponding IAC with a balance factor of *s* = 0.5 for verification. Across all test cases, the predicted spectral phase closely follows the ground truth, and the reconstructed IAC signal exhibits excellent agreement with the synthesized input signal. These results indicate that the model does not memorize specific spectral patterns but instead learns a physically consistent mapping between the IAC signal and the underlying spectral phase of the electric field.

**FIGURE 3:**
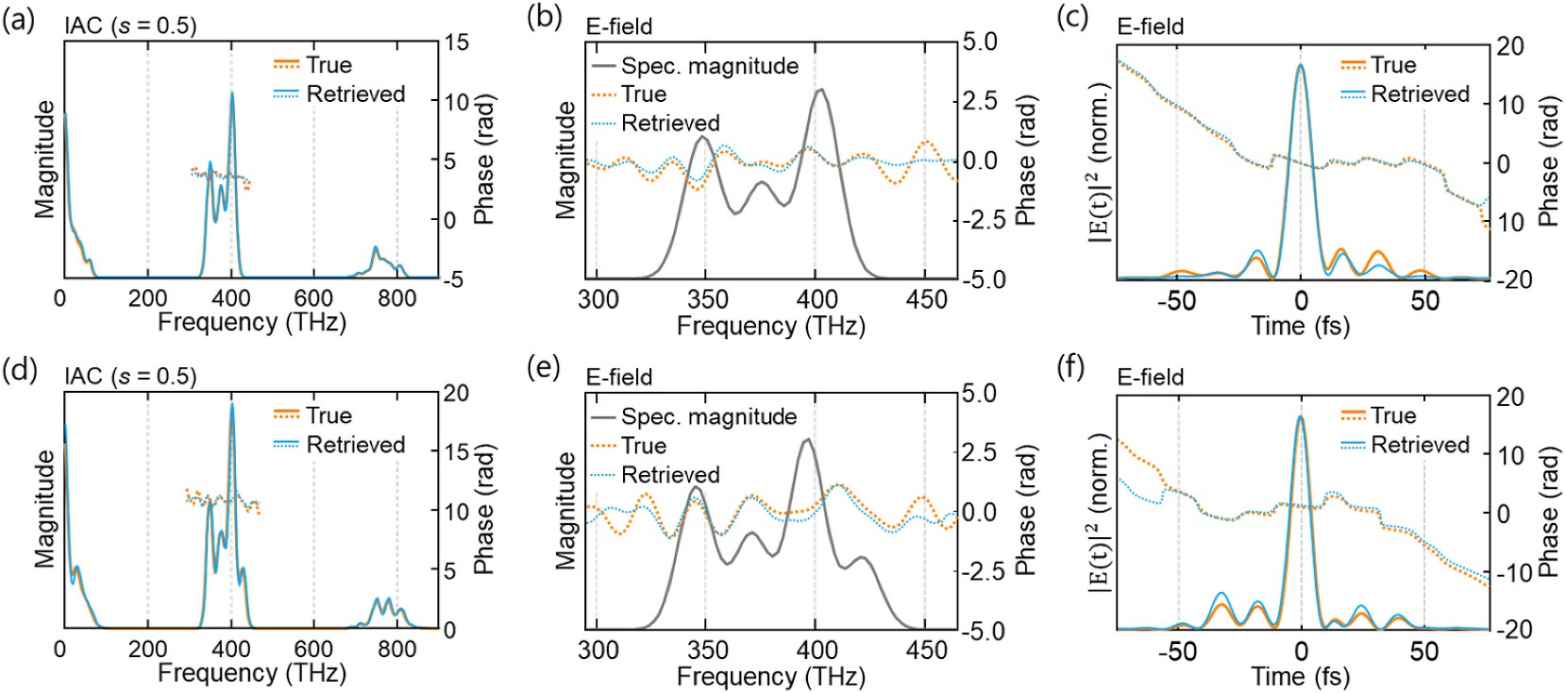
Comparison between the ground truth and the DeepPENGUIN predictions. Representative test cases were generated using different numbers of Gaussian spectral peaks combined with a random phase based on a Fourier series: (a–c) three peaks, (d–f) four peaks. For each case, the complex electric field in the frequency domain was used to calculate the IAC signal with *s* = 0.5, which served as the input to the DeepPENGUIN model together with the multi‐peak power spectrum. (a, d) Frequency‐domain representation of the true (orange) and retrieved (blue) IAC spectra, where magnitudes are shown as solid lines and phases as dotted lines (flat phase sections are omitted). (b, e) True (orange) and retrieved (blue) spectral phase of the electric field in the frequency domain, and the reference spectral magnitude of the pulse (gray). (c, f) Time‐domain representation of the retrieved fields in (b, e) showing the intensity envelopes (solid lines) and temporal phases (dotted lines).

### 3.2 Validation With Experimental Data

To verify the DeepPENGUIN model's performance not only for parametrically generated pulse shapes but also for realistic ultrafast optical pulses, we used the measured electric field of dispersion‐minimized 8‐fs few‐cycle laser pulses from a Ti:sapphire oscillator with an 800‐nm center wavelength as the basis for validation. These pulse data were not used during training. First, the true electric field of these pulses was independently measured using a well‐established SHG‐FROG measurement setup and a ptychographic FROG retrieval algorithm [44, 45, 46] under dispersion‐minimized conditions (see Supporting Information S1: Section 3 and Figure S3). The IAC signal of this reference field was computed using a balance factor of *s =* 0.5 and subsequently supplied as an input to DeepPENGUIN together with the reference power spectrum of the laser pulse. The predicted spectral phase was then combined with the reference power spectrum to obtain the reconstructed electric field, from which the corresponding reconstructed IAC signal was calculated for a direct comparison between the true field and the prediction. As shown in Figure 4a,b, the retrieved IAC signal in both the time and frequency domains, respectively, exhibits good agreement with the input signal. Figure 4c,d depict the reconstructed electric field in the frequency and time domains, respectively, showing a close match with the true data. This confirms that DeepPENGUIN can successfully reconstruct the spectral phase of a realistic few‐cycle ultrashort pulse.

**FIGURE 4:**
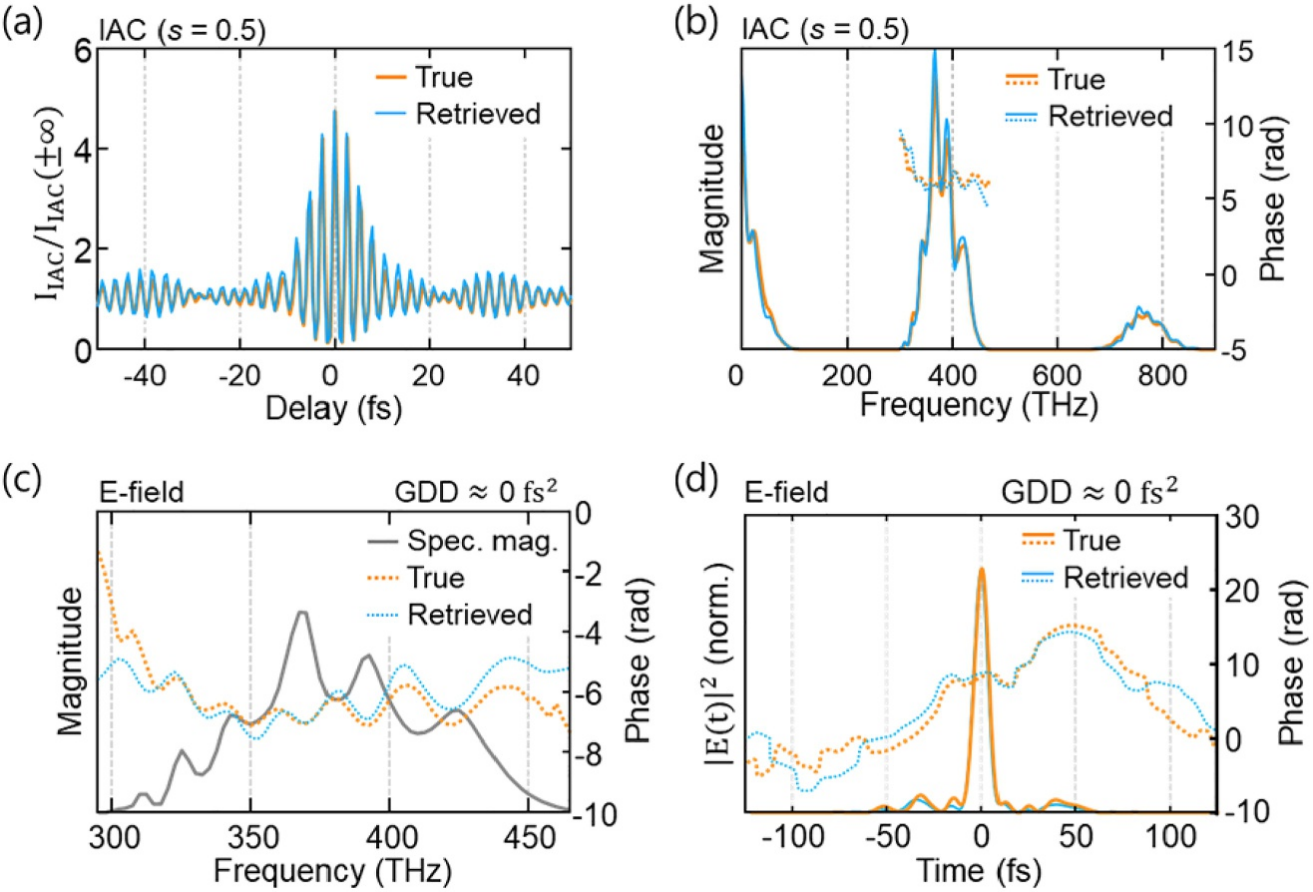
DeepPENGUIN retrieval results with experimental data. (a) True (orange) and DeepPENGUIN retrieved (blue) time‐domain IAC signals. (b) Frequency‐domain representation of the true (orange) and retrieved (blue) IAC signals with magnitudes shown as solid lines and phases as dotted lines (flat phase sections are omitted). (c) True (orange) and retrieved (blue) spectral phase of the electric field in the frequency domain, and the independently provided reference spectral magnitude of the laser pulse (gray). (d) Time‐domain representation of the true (orange) and retrieved (blue) fields in (c) showing the intensity envelopes (solid lines) and temporal phases (dotted lines).

### 3.3 Validation of GDD Robustness

The GDD robustness of DeepPENGUIN was validated using experimentally retrieved electric fields obtained from SHG‐FROG measurements reconstructed with a ptychographic FROG retrieval algorithm (Figure 5a,b). Four dispersion conditions were experimentally recorded by inserting fused‐silica plates with thicknesses of 0.3, 0.6, 1.6, and 2.0 mm. For each case, the complex electric field retrieved by ptychographic FROG (orange dots) was used to generate the corresponding IAC trace with a balance factor of *s* = 0.5, and the reference spectral magnitude (gray) was taken directly from the retrieved field. Note that residual third‐order dispersion was removed from the dispersed electric fields measured by FROG which served as the ground truths for the DeepPENGUIN validation. Since the deep‐learning model was trained specifically to infer the GDD over a wide range, higher dispersion orders were modeled in the training data only within a limited range via a Fourier series, as described above, to reproduce arbitrary spectral phase shapes of typical ultrashort pulses near the Fourier transform limit. DeepPENGUIN predictions of the spectral phase (blue dots) were then compared with these ground truths (Figure 5a,b).

**FIGURE 5:**
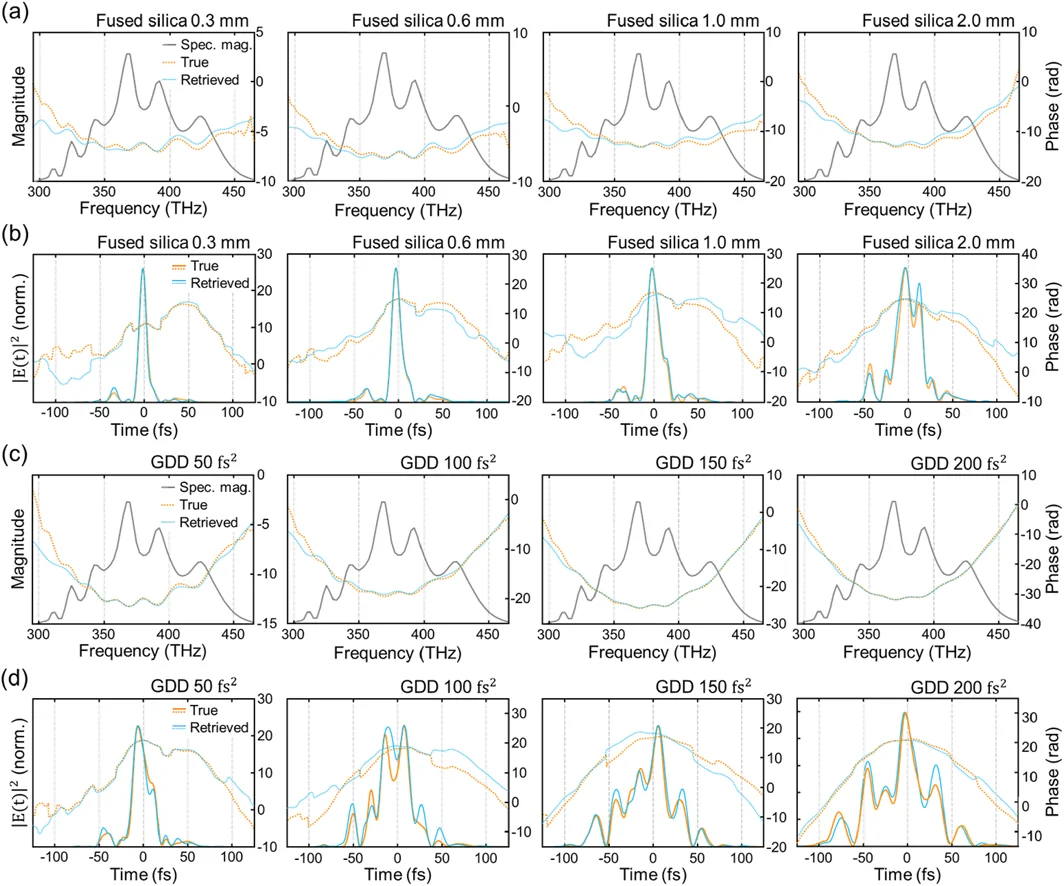
DeepPENGUIN retrieval results with experimental data under controlled dispersion conditions. (a) True (orange) and DeepPENGUIN (blue) predicted spectral phases measured after propagation through fused silica with thicknesses of 0.3, 0.6, 1.0, and 2.0 mm, corresponding to retrieved GDD values of 21.6, 43.2, 57.6, and 86.4 fs^2^, respectively (from left to right), and the independently measured reference spectral magnitude (gray). (b) Corresponding time‐domain representations of the electric fields in (a), showing the reconstructed temporal intensity envelopes (solid lines) and temporal phases (dotted lines). (c) Spectral phases of the reference dispersion‐minimized electric field (orange) with synthetically added GDD of 50, 100, 150, and 200 fs^2^ (from left to right), respectively, in comparison with the DeepPENGUIN predictions (blue), and the reference spectral magnitude (gray). (d) Corresponding time‐domain representations of the electric fields in (c), showing the reconstructed temporal intensity envelopes (solid lines) and temporal phases (dotted lines) for both the true and predicted electric fields.

In addition, we evaluated the capability of the model to reconstruct the spectral phase for substantially larger GDD values than those achieved via fused‐silica insertion by numerically adding 50, 100, 150, and 200 fs^2^ of GDD (i.e., parabolic spectral phase) to the spectral phase of the dispersion‐minimized experimental reference field. The corresponding IAC signals were calculated from these artificially chirped electric fields and supplied to DeepPENGUIN together with the reference power spectrum. The retrieved spectral phases of the dispersed electric fields, as shown in Figure 5c,d, again matched the ground truth closely, demonstrating that DeepPENGUIN maintains consistent performance across a broad range of dispersion conditions. In the high‐dispersion regime, better results are achieved by training the DeepPENGUIN model independently for different amounts of constant GDD, spaced by 10–50 fs^2^, over a range of 0–200 fs^2^. For the DeepPENGUIN reconstruction, the amount of GDD is first roughly inferred from the supplied IAC signal, then the appropriate model corresponding to the inferred GDD value is selected, as described in Supporting Information S1: Section 4.

### 3.4 Evaluation of Noise Robustness

To quantitatively evaluate the robustness of the DeepPENGUIN framework in the presence of noise, its performance was compared with ptychographic FROG reconstruction over a broad range of noise levels. Noise was introduced to the simulated noise‐free measurement data to mimic physically realistic degradation of experimentally acquired signals. For each noise condition, equivalent random noise was applied to both the two‐dimensional SHG‐FROG spectrogram and the unbalanced‐intensity IAC trace, enabling a direct quantitative comparison between the two methods. The noise level is quantified using the contrast‐to‐noise ratio (CNR), which measures noise strength relative to the signal contrast. This definition is essential because SHG‐FROG and IAC encode phase information in fundamentally different ways. The SHG‐FROG spectrogram is intrinsically background‐free and is typically normalized such that its maximum signal intensity is unity. The spectrally resolved signal intensity at each delay time provides the information required for phase retrieval. Consequently, its CNR is equivalent to the conventional signal‐to‐noise ratio. In contrast, the time‐domain IAC signal contains a constant background, and its phase information is directly encoded in the interferometric modulation with respect to that background rather than in the absolute signal amplitude. To account for this difference, noise in the IAC signal is scaled with respect to the peak‐to‐background modulation amplitude, ensuring that a given CNR corresponds to the same relative degradation of phase‐encoding features for both techniques. Additive normal‐distributed Gaussian white noise, n(τ) and N(ω,τ), was introduced to the normalized IAC traces and SHG‐FROG spectrograms, respectively, with a controlled standard deviation $\sigma_{\text{noise}}$ determined by the respective CNR, which is defined as:

(10) $$\begin{array}{c} {\text{CNR}}_{\text{IAC}}=\frac{A_{\text{contrast}}}{\sigma_{\text{noise}}},{\text{CNR}}_{\text{FROG}}=\frac{1}{\sigma_{\text{noise}}} \end{array},$$

where $A_{\text{contrast}}$ denotes the peak‐to‐background modulation amplitude of a noise‐free IAC measurement, defined as $A_{\text{contrast}}=I_{\text{IAC}}\left(0\right)/I_{\text{IAC}}\left(\pm \infty \right)$ according to Equation (1). The IAC and FROG signals affected by noise are thus given by:

(11) $$\begin{array}{c} I_{\text{IAC}}^{\text{noisy}}\left(\tau \right)=I_{\text{IAC}}^{\text{clean}}\left(\tau \right)+\frac{A_{\text{contrast}}}{{\text{CNR}}_{\text{IAC}}}n\left(\tau \right) \end{array},$$

(12) $$\begin{array}{c} I_{\text{FROG}}^{\text{noisy}}\left(\omega ,\tau \right)=I_{\text{FROG}}^{\text{clean}}\left(\omega ,\tau \right)+\frac{1}{{\text{CNR}}_{\text{FROG}}}N\left(\omega ,\tau \right) \end{array}.$$

This definition ensures that a given CNR corresponds to an equivalent relative noise strength with respect to the phase‐encoding modulation depth in both the one‐dimensional IAC and the two‐dimensional SHG‐FROG spectrogram, enabling a physically consistent quantitative comparison of the noise robustness between ptychographic FROG and DeepPENGUIN.

The CNR was logarithmically swept from 500 down to 10 to reflect the exponential dependence of the noise amplitude on contrast. Reconstruction accuracy was assessed in terms of the mean absolute error (MAE) between the retrieved signal and the noise‐free reference signal (see Figure 6a). The comparison results demonstrate that the DeepPENGUIN model exhibits comparable or even better noise robustness than the ptychographic FROG algorithm which is recognized for its noise robustness. Ptychographic FROG shows a gradual increase of the MAE with decreasing CNR, where the retrieved spectral intensity steadily degrades as the CNR decreases, and the spectral phase reconstruction exhibits noticeable deterioration around CNR ≈ 200. Note that using the reference power spectrum as a constraint in the FROG reconstruction does not improve the MAE of the retrieved spectral phase. The DeepPENGUIN model, on the other hand, maintains consistently low MAE of the spectral phase down to approximately CNR ≈ 50, below which gradual degradation emerges. These observations confirm that DeepPENGUIN achieves comparable or slightly superior noise robustness relative to ptychographic FROG for phase retrieval. Representative reconstruction results are shown in Figure 6b–d. Figure 6b depicts a noise‐free and a noisy (CNR = 20) SHG‐FROG spectrogram, and the corresponding reconstructed spectrogram for the noisy case. Figure 6c shows noise‐free and noisy (CNR = 20) IAC signals in the frequency domain. Using these noisy data with CNR = 20, the frequency‐domain electric fields retrieved with DeepPENGUIN and with the ptychographic FROG algorithm are shown in Figure 6d. Here, DeepPENGUIN reproduces the true spectral phase reasonably well, whereas the ptychographic FROG retrieval exhibits noticeable phase deviations. These results highlight the strong noise robustness of the DeepPENGUIN framework.

**FIGURE 6:**
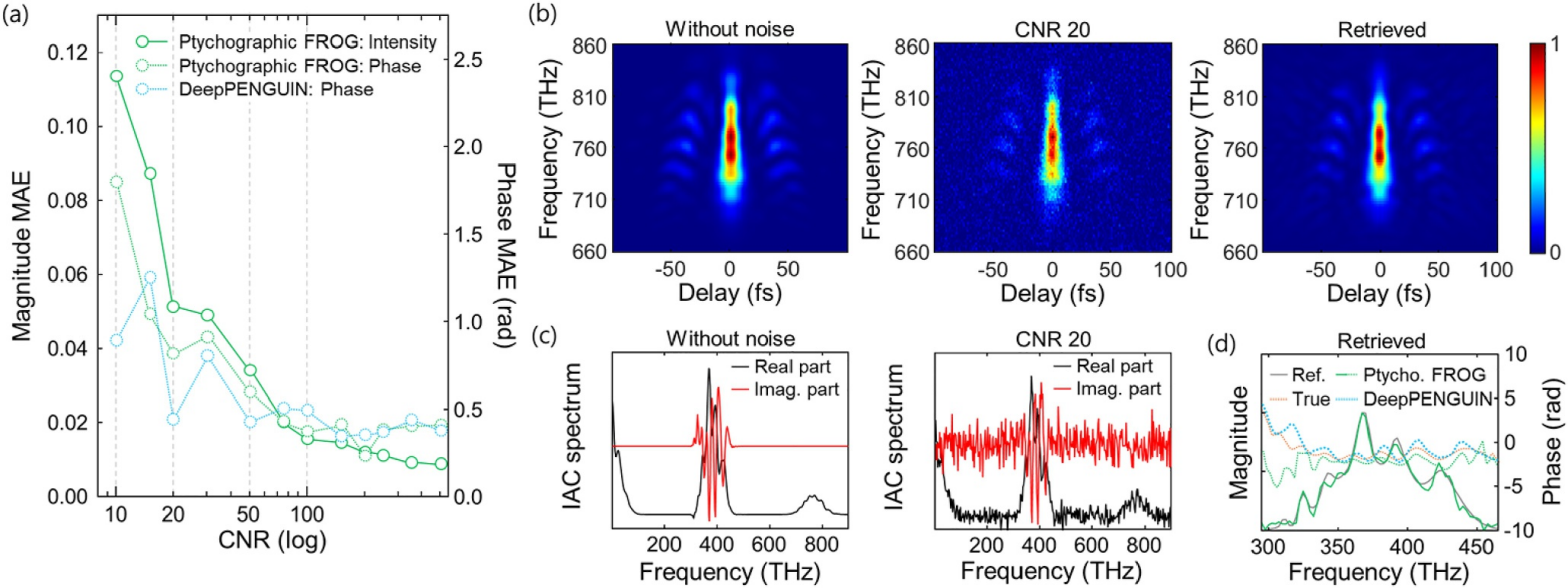
Noise robustness comparison between electric field reconstructions using ptychographic FROG and DeepPENGUIN. (a) Mean absolute error (MAE) of the spectral intensity (green solid) and phase (green dotted) retrieved by FROG in comparison with the phase retrieved by DeepPENGUIN (blue dotted) as a function of CNR (logarithmic scale). (b) Noise‐free SHG‐FROG spectrogram (left), CNR = 20 spectrogram (middle), and the CNR = 20 ptychographic reconstruction (right). (c) Real (black) and imaginary part (red) of the IAC spectrum without noise (left) and with CNR = 20 (right). (d) Retrieved electric field in the frequency domain at CNR = 20. Reference spectral magnitude (gray) and phase (orange dotted), reconstructed magnitude (green solid) and phase (green dotted) with ptychographic FROG and via DeepPENGUIN (blue dotted).

## 4 Conclusion

This work demonstrates that the spectral phase of ultrafast optical pulses can be robustly reconstructed from an unbalanced‐intensity nonlinear IAC signal using a data‐driven approach without relying on spectrally resolved detection or iterative phase retrieval. By learning the physical mapping between the frequency‐domain IAC signal and the underlying spectral phase, DeepPENGUIN overcomes the limitations of the original numerical PENGUIN algorithm [37] in the strongly dispersed regime. A key feature of the proposed framework is the explicit incorporation of physics‐guided constraints at multiple levels. First, a mathematical consistency constraint is introduced through an additional loss term, in which the predicted complex electric field is used to reconstruct the interferometric autocorrelation signal, and consistency with the measured IAC is enforced during training. Second, a spectral constraint is imposed using the power spectrum of the electric field, ensuring experimental consistency of the reconstructed spectral magnitude and allowing the network capacity to focus primarily on phase inference. Third, the spectral phase is represented using cosine and sine components (Cartesian rather than polar representation), which enforces phase continuity and avoids ambiguities associated with direct phase regression. Finally, a multi‐resolution convolutional architecture is employed to capture both local spectral modulations and long‐range spectral correlations, enabling robust learning across diverse pulse shapes and dispersion conditions. Together, these design choices lead to stable and accurate spectral phase reconstruction over a broad range of pulse durations and dispersion regimes.

Experimental validation using few‐cycle Ti:sapphire pulses confirms that DeepPENGUIN accurately retrieves the spectral phase under realistic conditions. Furthermore, a quantitative noise robustness analysis based on a physically consistent CNR metric shows that the performance of DeepPENGUIN is comparable to that of ptychographic FROG. It has been previously established that the PENGUIN method performs best for dispersion‐minimized pulses near the Fourier‐transform limit and supports at least octave‐broad spectra; however, it is important to note that the reconstruction accuracy is fundamentally limited by dispersion [37]. A large amount of dispersion such as GDD narrows the bandwidth of the fundamental IAC peak with respect to the spectral bandwidth of the field, thus leading to signal loss (both magnitude and phase information) at the spectral edges and increasing retrieval error. Artificially extremely dispersed pulses for special applications requiring simultaneously a broad spectrum and long pulse duration are therefore difficult to accurately reconstruct via PENGUIN. Nonetheless, the DeepPENGUIN framework significantly extends the dispersion limit for useful pulse reconstruction compared to the previous numerical algorithm by employing data‐driven inference based on the information contained within all harmonic peaks of the IAC signal as well as the spectral constraint.

As a proof of principle, the experimental validation in this work focuses on GDD as the dominant dispersion order, whereas higher orders are modeled as a Fourier series with lower strength, which is reflected in the choice of training data. Including higher dominant dispersion orders is straight‐forward by incorporating higher‐order phase terms into the training dataset [47]. Notably, applying 200 fs^2^ of GDD to an 8‐fs Gaussian pulse increases the pulse duration by almost a factor of 9 and constitutes a dispersion regime far from the Fourier‐transform limit. The demonstrated high retrieval quality in this regime suggests the applicability of DeepPENGUIN for a wide range of dispersion conditions and pulse shapes in practical ultrafast optics applications that do not require extreme dispersion. For integrated photonic applications, where typically narrowband sub‐picosecond pulses in the telecommunication wavelength range are used, material dispersion is commonly undesired but some dispersion is unavoidable due to waveguide propagation. In this moderate‐dispersion regime, DeepPENGUIN can provide a valuable, fully integrated metrology tool owed to its ability to retrieve the complete electric field from a simple autocorrelation measurement without spectrally resolved detection. The significantly reduced system complexity and computational overhead is particularly relevant for integrated photonic platforms where dispersion management, system compactness, and real‐time operation impose stringent constraints. DeepPENGUIN addresses these requirements by enabling spectrometer‐free and non‐iterative ultrafast pulse diagnostics, thus facilitating adaptive control and feedback stabilization in integrated photonic systems.

Although a universal solver for the PENGUIN phase retrieval problem remains beyond the scope of this study due to the fundamental dispersion limitation, the demonstrated numerical and experimental results establish a reliable reconstruction framework across a broad range of practical pulse conditions far from the Fourier‐transform limit. Importantly, the role of deep learning in this work is fundamentally different from its use in many previous pulse characterization studies. Rather than accelerating an already solvable reconstruction problem, DeepPENGUIN enables practical inversion of a phase‐preserving one‐dimensional IAC measurement for which no general closed‐form retrieval framework currently exists. This distinction highlights a broader opportunity for computational photonics: machine learning can unlock the potential of experimentally simple measurement implementations that would otherwise remain analytically inaccessible.

## Author Contributions

**Tae‐In Jeong:** conceptualization, methodology, software, writing – original draft. **Eunji Choi:** methodology, software. **Mengkun Liu:** writing – review and editing, supervision. **Seungchul Kim:** conceptualization, project administration, writing – review and editing, supervision, funding acquisition. **Alexander Gliserin:** conceptualization, project administration, writing – review and editing, supervision.

## Conflicts of Interest

The authors declare no conflicts of interest.

## Supporting information


Supporting Information S1

## Data Availability

The data that support the findings of this study are available from the corresponding author upon reasonable request.
