# Supplementary material for: Deep Learning Enabled Ultrafast Optical Spectral Phase Retrieval via Unbalanced‐Intensity Autocorrelation
Source: Nanophotonics. 2026 May 21;15(10):e70144. doi: 10.1002/nap2.70144 (PMC13642475; doi:10.1002/nap2.70144)
Supplement: Supplementary file 1 [file NAP2-15-e70144-s001.pdf]

Deep learning enabled ultrafast optical spectral phase retrieval via unbalanced-intensity autocorrelation

Tae-In Jeong[1,2,3], Eunji Choi[2], Mengkun Liu[3,4], Seungchul Kim[1,2,a)], Alexander Gliserin[1,2,a)]

AFFILIATIONS

[1] *Department of Opto-mechatronics engineering, Pusan National University, Busan 46241, Republic of Korea.*

[2] *Department of Cogno-mechatronics engineering, Pusan National University, Busan 46241, Republic of Korea.*

[3] *Department of Physics and Astronomy, Stony Brook University, Stony Brook, New York, USA.*

[4] *National Synchrotron Light Source II, Brookhaven National Laboratory, Upton, New York, USA*

[a)] Author to whom correspondence should be addressed: s.kim@pusan.ac.kr, alex@mpk.or.kr

1. Harmonic expression of the unbalanced-intensity nonlinear IAC in the frequency domain

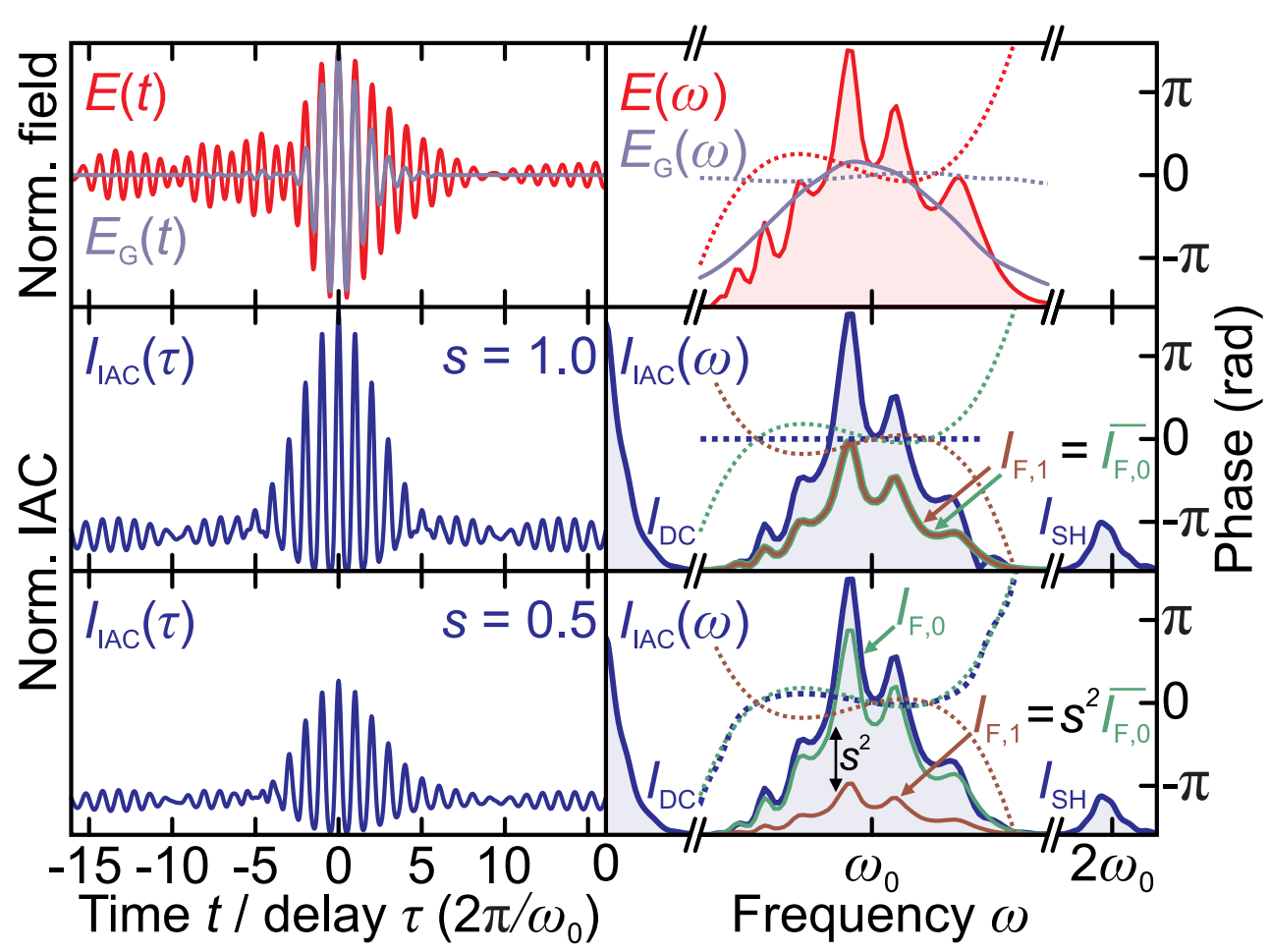

FIG. S1. Principle of phase-preserving unbalanced-intensity IAC for PENGUIN. Exemplary few-cycle pulse (red) and its IAC (dark blue) in the time and frequency domains. Spectral magnitudes are shown as solid lines and phases as dotted lines in the right panels. The fundamental-frequency $I_{F,0}$ (green) and $I_{F,1}$ (brown) components are complex conjugate products of the fundamental field (red) and the gating field (purple) in the frequency domain scaled by a factor of s^2 according to Eqs. (S3) and (S4). For $s = 1$, $I_{F,0}$ and $I_{F,1}$ are exact complex conjugates, thus their spectral phases cancel out after summation resulting in a time-symmetric IAC signal. For $s < 1$, spectral phase information is preserved in the IAC signal after summation of $I_{F,0}$ and $I_{F,1}$ by breaking its time-reversal symmetry.

The unbalanced-intensity nonlinear interferometric autocorrelation (IAC) signal, as defined in Eq. (1) in the main manuscript, can be expressed in the frequency domain as a sum of spectral components at distinct harmonic orders[1]. After evaluating the binomial and magnitude-squared operations, the time-domain IAC integral consists of cross-correlation terms between different powers of $E(t)$. In the frequency domain, these integral terms can be replaced by simple products of the field powers according to the convolution theorem, which significantly simplifies the expression for the IAC signal. Ignoring negative-frequency and delay-independent terms which contribute only a constant background, the background-free unbalanced-intensity second-order IAC signal is expressed in the frequency domain as:

$$I_{\mathrm{IAC}}^{(2)}(\omega) = I_{\mathrm{DC}}(\omega) + I_{\mathrm{F,0}}(\omega) + I_{\mathrm{F,1}}(\omega) + I_{\mathrm{SH}}(\omega), \quad \text{(S1)}$$

$$I_{\mathrm{DC}}(\omega) = 4s^2|\mathcal{F}\{|E(t)|^2\}|^2, \quad \text{(S2)}$$

$$I_{\mathrm{F,0}}(\omega) = 2s\mathcal{F}\{E(t)\}\overline{\mathcal{F}\{|E(t)|^2E(t)\}}, \quad \text{(S3)}$$

$$I_{\mathrm{F,1}}(\omega) = 2s^3\mathcal{F}\{|E(t)|^2E(t)\}\overline{\mathcal{F}\{E(t)\}} = s^2\overline{I_{F,0}(\omega)}, \quad \text{(S4)}$$

$$I_{\mathrm{SH}}(\omega) = s^2|\mathcal{F}\{E(t)^2\}|^2, \quad \text{(S5)}$$

with $\mathcal{F}$ denoting Fourier transformation from the time domain into the frequency domain, and the top bar complex conjugation. This derivation is schematically depicted in Fig. S1, showing an exemplary few-cycle pulse and the resulting balanced and unbalanced-intensity IAC with their harmonic components in the time and frequency domains. The I_{DC} and I_{SH} components are magnitude-squared values and therefore contain no spectral phase information. The fundamental-frequency $I_{\mathrm{F,0}}$ and $I_{\mathrm{F,1}}$ components are products between the fundamental electric field and its power, $E_{\mathrm{G}}(t) = |E(t)|^2E(t)$, referred to as gating field (see Fig. S1, top panels), with a non-trivial spectral phase. In a balanced IAC ($s = 1$), $I_{\mathrm{F,0}}$ and $I_{\mathrm{F,1}}$ are exact complex conjugates, therefore their spectral phase cancels out resulting in a time-symmetric IAC after summation (see Fig. S1, middle panels). However, a balance factor of $s < 1$ breaks this time-reversal symmetry since $I_{\mathrm{F,0}}$ and $I_{\mathrm{F,1}}$ are scaled by a factor of s^2. In this case, the spectral phase does not cancel out after summation in Eq. (S1), which preserves spectral phase information in the resulting IAC signal (see Fig. S1, bottom panels) and enables field retrieval for low-dispersion pulses by numerically solving Eq. (S3) for the fundamental field with an iterative algorithm[1].

2. Architectural details of the DeepPENGUIN model

The DeepPENGUIN model is designed to infer the spectral phase of ultrafast optical pulses from unbalanced-intensity IAC measurements while explicitly incorporating physical constraints of the optical field. Beyond the overall encoder structure described in the main text, several architectural choices were made to ensure stable learning, phase continuity, and physical interpretability. First, all convolutional and fully connected layers employ the Swish activation function[2]. Unlike linear activation such as ReLu, Swish provides a smooth and non-monotonic nonlinearity with continuous gradients over the entire input domain. This property is particularly advantageous for spectral phase learning, where the target phase function $\phi(\omega)$ varies continuously with frequency. Smooth gradient propagation suppresses artificial phase discontinuities and high-frequency oscillations, thereby improving convergence stability and phase continuity across the spectrum. Each encoder stage consists of a multi-resolution convolution (MRC) block followed by a down-sampling convolution (DSC) block[3-5]. In the MRC block, multiple one-dimensional convolutional paths with different kernel sizes operate in parallel, enabling simultaneous extraction of spectral features at multiple frequency scales. Narrow kernels capture local spectral modulations associated with fine interference features in the IAC spectrum, while wider kernels encode long-range spectral correlations arising from broadband temporal interference. Down-sampling is implemented using strided convolutions rather than pooling operations. Strided convolutions reduce the spectral resolution while preserving phase-sensitive information through learnable filters, whereas pooling operations may discard subtle spectral features critical for accurate phase reconstruction. As the network depth increases, the DSC blocks progressively compress the spectral dimension and expand the channel dimension, allowing the network to form compact yet expressive representations of the spectral phase information. The spectral magnitude of the reconstructed electric field is structurally enforced using the independently measured power spectrum, $S(\omega) = |E(\omega)|^2$. Explicitly combining the predicted phase representation with the measured spectral magnitude allows the model capacity to be fully devoted to phase inference. This structural constraint

improves generalization and robustness, particularly under noisy experimental conditions. Layer normalization is employed in the fully connected layers to stabilize training by normalizing feature activations within each individual sample rather than across the batch[6]. This avoids mixing statistical information from spectrally distinct pulse conditions and preserves the relative structure of broadband spectral representations, which is critical for accurate phase inference. Dropout is selectively applied at the fully connected latent layer that integrates the convolutional features before phase prediction[7]. This regularizes global spectral representations while preserving physically meaningful local phase correlations learned in the convolutional encoder. These architectural choices collectively ensure the DeepPENGUIN model remains both expressive and physically consistent, enabling accurate and stable phase reconstruction without iterative optimization.

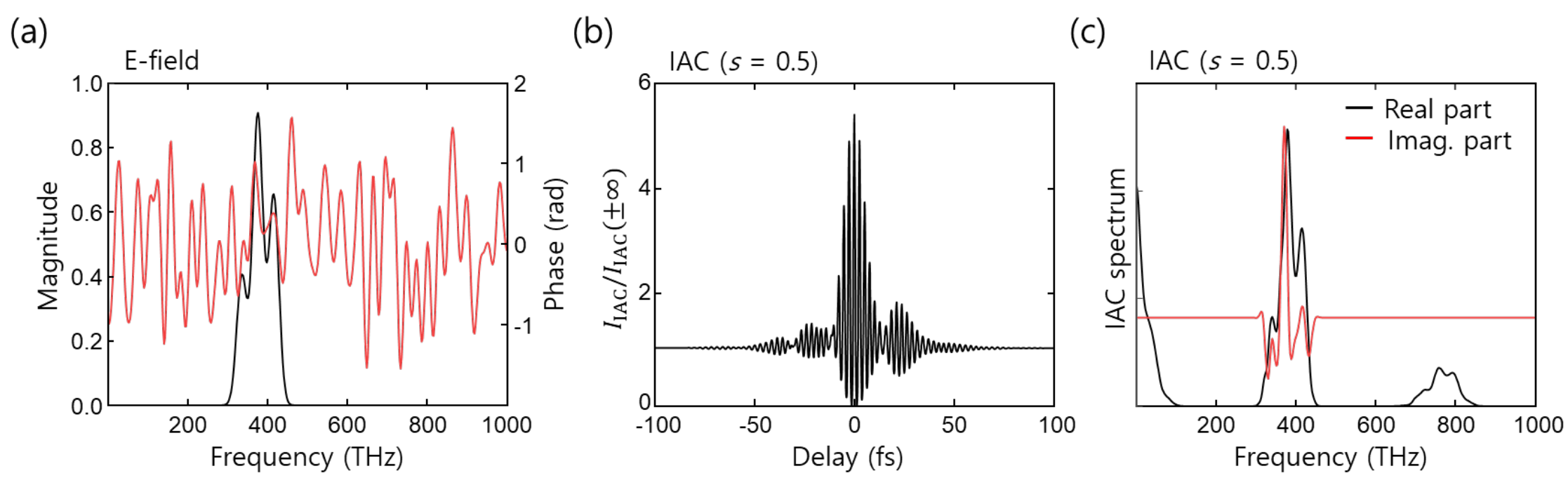

FIG. S2. Synthetic training example used for DeepPENGUIN dataset generation. (a) Example of a spectrally engineered electric field consisting of three Gaussian spectral peaks with different relative amplitudes, together with a Fourier-series based phase function generated using a dominant mode parameter of $K = 400$. The magnitude (black) and phase (red) are defined in the frequency domain constituting the complex electric field. (b) Corresponding calculated unbalanced-intensity ($s = 0.5$) IAC signal in the time domain. (c) Frequency-domain representation of the time-domain IAC in (b) obtained via Fourier transformation, showing the normalized real (black) and imaginary parts (red) used together with the spectral magnitude in (a) as neural network inputs during supervised learning.

3. SHG-FROG measurement and ptychographic FROG retrieval algorithm

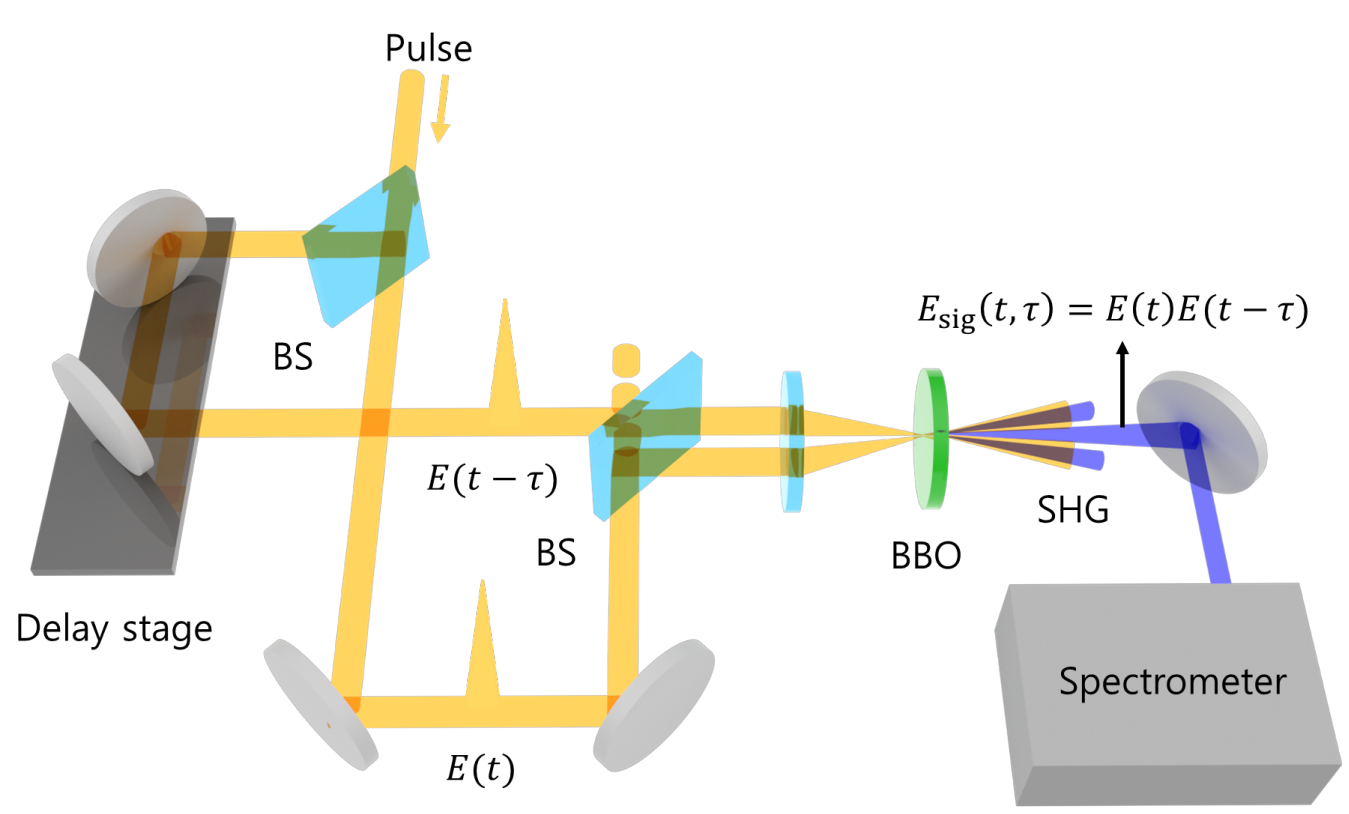

FIG. S3. Schematic of the noncollinear SHG-FROG setup based on a Mach-Zehnder interferometer geometry. The incident pulse is split into two replicas using a beam splitter. One beam is delayed by a motorized translation stage and both beams are focused into a thin BBO crystal at a small crossing angle. Inside the crystal, three second-harmonic beams are generated: two from the individual beams and one central beam from the overlap between their temporal intensity envelopes. The central beam is spatially filtered and directed into a spectrometer for frequency-resolved detection as a function of the delay.

To evaluate the spectral phase retrieval performance of the DeepPENGUIN model with a realistic ultrafast optical pulse, an additional pulse characterization measurement was performed using the well-established FROG method[8, 9]. In the SHG-FROG setup (see Fig. S3), the pulse was split into two arms, recombined at a non-collinear angle of approximately 10°, and focused onto a 20-μm-thick BBO crystal for type-I second-harmonic generation (SHG). The SHG signal produced at the temporal overlap between the two beams was recorded as a function of delay with a 1-fs step size using a spectrometer. The non-uniform spectral response arising from the BBO's phase-matching bandwidth and spectrally non-uniform detector efficiency was corrected by matching the frequency marginal of the FROG spectrogram to the auto-convolution of the independently measured fundamental power spectrum. The spectrally corrected SHG-FROG spectrogram was then supplied as the input to a ptychographic field retrieval algorithm[10]. The spectral phase of the electric field retrieved by ptychographic SHG-FROG is shown, for example, in Fig. 4c in the main manuscript as the ground truth for the DeepPENGUIN validation.

4. Automatic GDD inference and model selection

A major limitation of the original numerical PENGUIN algorithm[1] is the critical GDD limit, where strong dispersion constrains the spectral bandwidth of the IAC signal leading to unstable retrieval due to signal loss at the spectral edges of the electric field. Deep learning based approaches can mitigate the critical GDD limitation of the original PENGUIN algorithm by eliminating the constraint on the spectral bandwidth of the retrieved field imposed by the spectral narrowing of the IAC signal at high GDD conditions. In principle, a single deep learning model could be trained to cover the entire desired GDD span. However, constructing such a unified model requires combining training data from all dispersion regimes, which results in an extremely large dataset and necessitates a significantly enlarged network architecture. This leads to substantial increases in training time, memory requirement, and overall computational cost, making the unified-model approach impractical in terms of efficiency. To address this scalability issue, DeepPENGUIN adopts a multi-model strategy in which separate networks are individually trained for discrete GDD levels (0, 10, 20, 30, 40, 50, 60, 70, 80, 100, 150, and 200 fs²). The current implementation covers a dispersion range from 0 to 200 fs², while the framework is readily extendable to additional GDD levels if required. Each model is trained using a compact dataset confined to its assigned GDD level, which significantly reduces training complexity and memory usage. To enable automated selection among these GDD-specific networks, DeepPENGUIN introduces a GDD inference stage before the actual phase prediction. The input consists of the frequency-domain IAC and the power spectrum, $S(\omega)$. The power spectrum is assigned a flat phase to generate a Fourier transform limited reference electric field, and the corresponding IAC trace is computed. Direct comparison of the reference and experimental IAC traces in the time domain is avoided due to sensitivity to interferometric fringes and ambiguities in the envelope width estimation. Instead, both traces are Fourier-transformed into the frequency domain, and only the 0-200 THz component is retained to isolate the DC band. The inverse Fourier transform of this filtered signal produces the time-domain DC-band IAC, which represents the fringe-free temporal intensity autocorrelation of the pulse. The full width at half maximum (FWHM) of the DC-band IAC provides a robust scalar indicator of temporal broadening. Numerical characterization shows that the dependence of the DC band's temporal width on GDD follows a simple quadratic form,

$$\tau_{\mathrm{DC}}^2(\mathrm{GDD}) \approx \tau_{\mathrm{DC,0}}^2 + A \cdot \mathrm{GDD}^2, \tag{S6}$$

where $\tau_{\mathrm{DC,0}}^2$ is the width of the DC band of the IAC when using a flat phase and A is a calibration coefficient determined by the spectrum of the pulse. This relationship is calibrated via the reference power spectrum, $S(\omega)$. The retrieved spectral magnitude is combined with a flat spectral phase, $\phi(\omega) = 0$, (see Fig. S4a) to numerically generate the flat-phase IAC spectrum shown in Fig. S4b. Applying a 0-200 THz low-pass filter isolates the DC band (see Fig. S4c), and subsequent inverse Fourier transformation yields the reference time-domain DC-band IAC trace as shown in Fig. S4d with an FWHM width of $\tau_{\mathrm{DC,ref}}^2$. The same procedure is applied to the experimentally measured IAC (see Fig. S4e), resulting in the DC-band IAC signal shown in Fig. S4f with a corresponding width of $\tau_{\mathrm{DC,meas}}^2$. To determine A, a range of GDD values is numerically assigned to the reference spectrum, $S(\omega)$, then the corresponding DC-band IAC widths, $\tau_{\mathrm{DC}}^2(\mathrm{GDD})$, are computed, and the resulting dependence is fitted using Eq. (S6). Once the coefficient A is calibrated, the

accumulated GDD can be inferred directly from a single measured DC-band IAC width by solving Eq. (S6) for the GDD:

$$\mathrm{GDD} = \sqrt{\frac{\tau_{\mathrm{DC,meas}}^2 - \tau_{\mathrm{DC,ref}}^2}{A}}. \tag{S7}$$

In practice, the procedure requires no iterative pulse retrieval. The experimentally measured DC-band IAC width is converted into a GDD estimate using the pre-calibrated factor A, after which the corresponding GDD-specific DeepPENGUIN model is automatically selected for the final reconstruction of the spectral phase. This enables robust field retrieval across a wide dispersion range, including conditions well beyond the critical GDD limit of the conventional numerical PENGUIN field reconstruction algorithm.

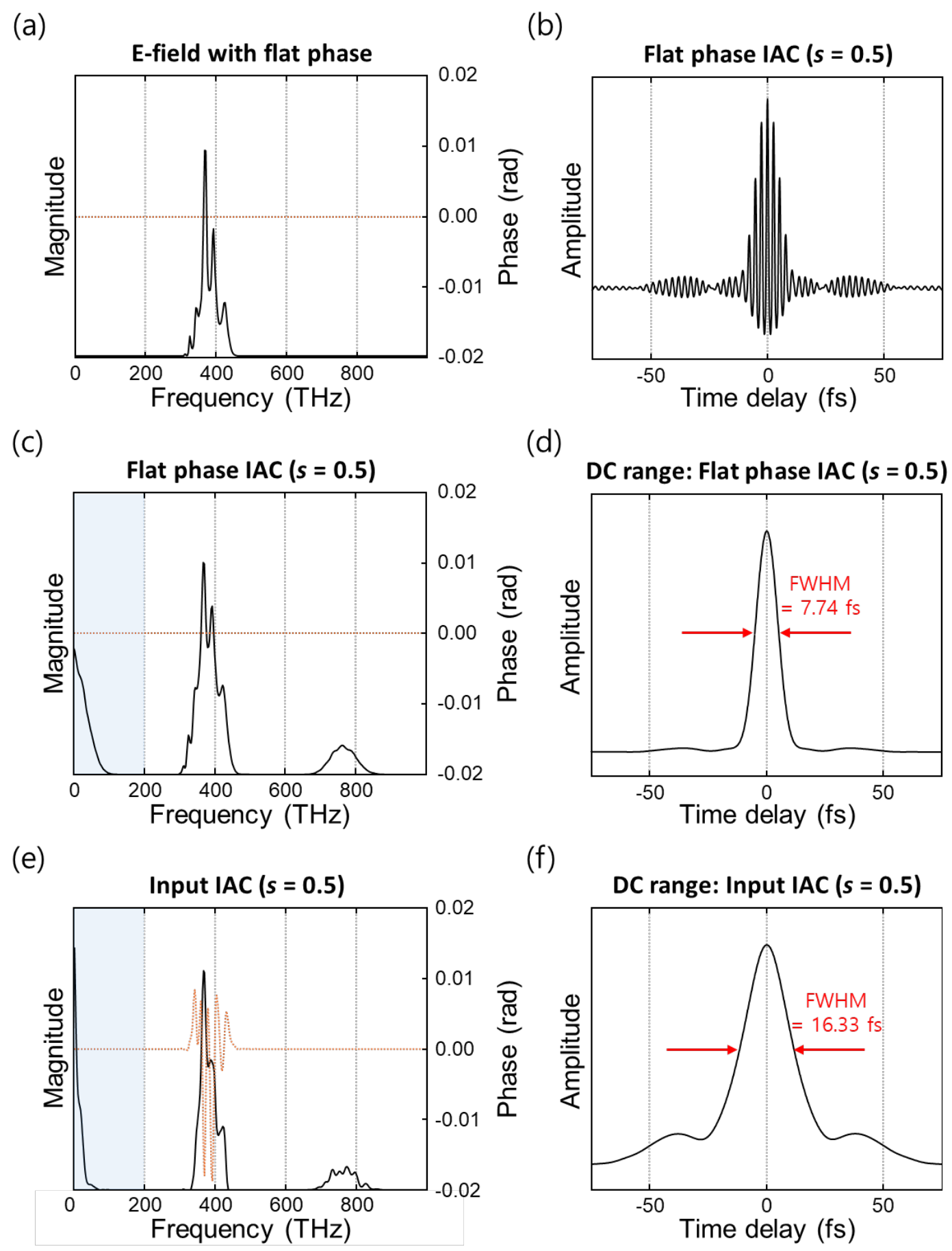

FIG. S4. Automatic GDD inference using the DC range of the IAC spectrum. (a) Frequency-domain representation of the electric field with a flat spectral phase, where the reference spectral magnitude, $\sqrt{S(\omega)}$, is shown as a black line and the flat phase as an orange dotted line. (b) Time-domain IAC trace calculated from the reference electric field with a flat phase in (a). (c) Frequency-domain representation of the IAC trace in (b) with the filtered 0-200 THz DC range highlighted in blue. (d) Time-domain DC-range IAC signal (corresponding to the intensity autocorrelation) calculated by inverse Fourier transformation of the filtered DC range in (c) with an FWHM width of 7.74 fs. (e) Measured frequency-domain IAC used as input for DeepPENGUIN with the filtered DC range highlighted in blue. (f) Measured time-domain DC-range IAC signal obtained by inverse Fourier transformation of the filtered DC range in (e) with an FWHM width of 16.33 fs, which is used for GDD inference based on Eq. (S7).

References

1. A. Gliserin, S. H. Chew, S. Kim and D. E. Kim, "Complete characterization of ultrafast optical fields by phase-preserving nonlinear autocorrelation," *Light: Science & Applications* **11** (1), 277 (2022). https://doi.org/10.1038/s41377-022-00978-3.
2. P. Ramachandran, B. Zoph and Q. V. Le, "Searching for activation functions," *arXiv preprint arXiv:1710.05941*, (2017). https://doi.org/10.48550/arXiv.1710.05941
3. T. Zahavy, A. Dikopoltsev, D. Moss, G. I. Haham, O. Cohen, S. Mannor and M. Segev, "Deep learning reconstruction of ultrashort pulses," *Optica* **5** (5), 666–673 (2018). https://doi.org/10.1364/OPTICA.5.000666.
4. C. Szegedy, W. Liu, Y. Jia, P. Sermanet, S. Reed, D. Anguelov, D. Erhan, V. Vanhoucke and A. Rabinovich, "Going deeper with convolutions," *Proceedings of the IEEE conference on computer vision and pattern recognition* 1–9 (2015).
5. J. T. Springenberg, A. Dosovitskiy, T. Brox and M. Riedmiller, "Striving for simplicity: The all convolutional net," *arXiv preprint arXiv:1412.6806*, (2014). https://doi.org/10.48550/arXiv.1412.6806
6. J. L. Ba, J. R. Kiros and G. E. Hinton, "Layer normalization," *arXiv preprint arXiv:1607.06450*, (2016). https://doi.org/10.48550/arXiv.1607.06450
7. N. Srivastava, G. Hinton, A. Krizhevsky, I. Sutskever and R. Salakhutdinov, "Dropout: a simple way to prevent neural networks from overfitting," *The journal of machine learning research* **15** (1), 1929–1958 (2014).
8. R. Trebino, K. W. DeLong, D. N. Fittinghoff, J. N. Sweetser, M. A. Krumbügel, B. A. Richman and D. J. Kane, "Measuring ultrashort laser pulses in the time-frequency domain using frequency-resolved optical gating," *Review of Scientific Instruments* **68** (9), 3277–3295 (1997). https://doi.org/10.1063/1.1148286
9. K. W. DeLong, D. N. Fittinghoff and R. Trebino, "Practical issues in ultrashort-laser-pulse measurement using frequency-resolved optical gating," *IEEE Journal of Quantum Electronics* **32** (7), 1253–1264 (1996). https://doi.org/10.1109/3.517026.
10. P. Sidorenko, O. Lahav, Z. Avnat and O. Cohen, "Ptychographic reconstruction algorithm for frequency-resolved optical gating: super-resolution and supreme robustness," *Optica* **3** (12), 1320–1330 (2016). https://doi.org/10.1364/OPTICA.3.001320.
